# Improved biomedical term selection in pseudo relevance feedback

**DOI:** 10.1093/database/bay056

**Published:** 2018-07-02

**Authors:** Muhammad Nabeel Asim, Muhammad Wasim, Muhammad Usman Ghani Khan, Waqar Mahmood

**Affiliations:** 1 Al-Khawarzmi Institute of Computer Science, University of Engineering & Technology, GT Road, Lahore, Punjab, Pakistan; 2 Department of Computer Science and Engineering, University of Engineering and Technology, Lahore, Pakistan

## Abstract

Biomedical information retrieval systems are becoming popular and complex due to massive amount of ever-growing biomedical literature. Users are unable to construct a precise and accurate query that represents the intended information in a clear manner. Therefore, query is expanded with the terms or features that retrieve more relevant information. Selection of appropriate expansion terms plays key role to improve the performance of retrieval task. We propose document frequency chi-square, a newer version of chi-square in pseudo relevance feedback for term selection. The effects of pre-processing on the performance of information retrieval specifically in biomedical domain are also depicted. On average, the proposed algorithm outperformed state-of-the-art term selection algorithms by 88% at pre-defined test points. Our experiments also conclude that, stemming cause a decrease in overall performance of the pseudo relevance feedback based information retrieval system particularly in biomedical domain.

Database URL: http://biodb.sdau.edu.cn/gan/

## Introduction

Retrieving documents that match the user query is one of the foremost challenge in almost all information retrieval systems. Continuous increase in literature causes keywords mismatch problem between user query and retrieved documents (1). To retrieve documents by measuring similarity between user query and indexed documents is even more difficult in biomedical domain because genes, drugs and diseases may have numerous synonyms. For example, a user inputs a query containing keywords like ‘Medical Practitioner’ and corpus has only relevant documents however all the documents contain the words such as doctor, physician etc. It can be seen that all the terms of documents are conveying same information but these are named differently due to which mismatch problem will occur and these documents which are more relevant to the query as compared to others will not be retrieved. In order to tackle this problem local and global query expansion (QE) is used. In global QE, knowledge sources and dictionaries like (WordNet, PubMed) are used to generate candidate expansion terms (2).

In local QE, statistical information is used to find candidate expansion terms from corpus. In this approach, documents are retrieved based on user query and top k retrieved documents are considered relevant. To select candidate expansion terms from top retrieved documents, different term selection techniques like chi-square, information gain (IG), Kullback–Leibler divergence (KLD) and dice are used. It has been observed that the online available data has vividly increased in volume while the number of query terms is very scarce (3).

According to Lesk *et al*. average query length used to be 2.30 (4) words and it remained same even after 10 years (5). At present, there has been a rise in the trend of providing quite lengthy queries containing (five or more words), but still most common queries contain only couple of words (6). Therefore, the scope of QE has increased over the time. QE can also decrease the performance of information retrieval. In global QE candidate expansion terms extracted from dictionaries may cause decrease in performance due to word ambiguity problem. If we have a query like ‘Which bank provides more profit?’, to expand this query, we will find synonyms of query terms from dictionaries. In this query word ‘bank’ can be used in two different scenarios. It can be either used to refer financial institution or river bank. Therefore, in global QE word sense disambiguation in query words is mandatory. Lesk algorithm is used for word sense disambiguation (7).

In local QE all the retrieved documents against a particular user query are not relevant to the user query (8). This may lead to the imperfect and faulty terms pool (the pool of all terms present in top retrieved documents) that may contain many redundant and irrelevant terms. Expanding the query with such terms may even drift the query to retrieve irrelevant items (3). Hence idea behind the selection of candidate expansion terms from terms pool is to first remove these redundant or irrelevant terms from the term pool. Term selection for QE will allow only the selection of most relevant terms against particular user query. Therefore, these days term selection for QE is one of the hottest topics of research in the domain of information retrieval (9).

There are two major types of term selection methods for QE: (i) based on corpus statistics and (ii) based on term association. The choice of these methods depends on the document retrieval models e.g. Okapi BM25, TFIDF and Language Models (3). The selection methods based on term association are used to evaluate the goodness of terms based on their co-occurrence in the feedback documents. Whereas, selection methods based on corpus statistics are used to estimate the goodness of the terms based on their distribution in the corpus. In biomedical domain, it is still a huge challenge for researchers to develop an extraordinary performing term selection method for QE that must be able to outperform available methods with a very high edge (10).

Mostly widely used term selection method ‘Chi-Square’ suffers from document misclassification problem as its ability to select most affective and worthy terms for QE gets affected by the defined threshold of relevant and non-relevant class in pseudo relevance feedback. To tackle mentioned problem, we propose a new technique document frequency chi-square (DFC) and compare it with eight term selection algorithms including two different versions of chi-square proposed by Carpineto (11). Moreover, in biomedical domain effects of pre-processing on the performance of pseudo relevance feedback are also discussed. We used mean average precision (MAP) to evaluate the integrity of presented algorithm on TREC 2006 Genomic (12) dataset.

## Related work

Efficient information retrieval systems are required to get relevant information against particular user query from rapidly growing biomedical literature (13). A major concern in information retrieval system is the word mismatch problem in which the same concept may be described using semantically similar but having syntactically different from of terms in both query and documents (14). For example, user query may contain a phrase like ‘cure of depression’, but the corpus documents may have different yet semantically similar phrase like ‘depression treatment’. Both are referring to same concept with different words. This problem can be solved using two approaches: query paraphrasing and QE.

In query paraphrasing approach, query words are replaced by their synonyms in order to generate query paraphrases. In above example, ‘cure’ can be replaced by its synonym ‘treatment’ to generate the paraphrase ‘treatment of depression’. Generated paraphrases are then used to retrieve documents from corpus. Zukerman *et al*. used WordNet (15) and parts of speech information to find the synonyms for paraphrase generation. Their experiment revealed a reasonable improvement in the process of retrieving relevant documents despite having issues in part-of-speech (POS) tagging (16).

QE techniques can further be categorized as global and local techniques. In global QE, dictionaries and knowledge resources are used to find expansion terms (17). Chu *et al*. performed global QE by selecting the candidate expansion terms using knowledge resources of UMLS Meta-Thesaurus and Semantic Networks. They showed 33% improvement in performance of ohsumed dataset based 40 queries, by expanding these queries using domain specific knowledge resources and document retrieval models (18). On the other hand, Stokes *et al*. (19) used various biomedical knowledge resources like GO, EntrezGene, ADAM etc. to improve the overall performance of information retrieval system. They also claimed that the performance of information retrieval system (19) can be increased by focusing on two factors: choice of good document ranking algorithm; and use of domain specific knowledge resources.

One of the concerns with global QE is the fact that due to unstoppable progress in new discoveries and ongoing research, available knowledge resources are in constant need of update. However, it is difficult to update the available knowledge resources rapidly. Therefore, researchers of information retrieval community are focusing on improving the system using local QE. In this approach, user queries are provided to retrieval models (Okapi BM25, TFIDF) which rank the corpus documents by measuring similarity between queries and documents. Top K documents are labeled as relevant to user information. These retrieved documents are used to generate term pool which contains all terms present in relevant documents. Different techniques like chi-square, IG, KLD, CoDice etc. are used to select terms from generated term pool. Jagendra *et al*. improved the performance of local QE method by introducing an aggregation technique for term selection. They combined four term selection techniques [KLD, co-occurrence, Robertson selection value (RSV) and IG] using proposed aggregation method. In order to apply Borda combination technique, all the individual term selection methods are applied and lists of candidate terms are obtained from all the methods. These ranked lists are then used to select the final QE terms. Terms having highest aggregation score chosen as the final expansion terms. Jagendra *et al*. illustrated that some of the expansion terms caused query drift (20). In order to tackle this problem, they performed semantic filtering by applying word2vec approach and showed 2% improvement in results.

Some researchers are also looking for ways to combine both local and global QE techniques (21, 22). In this regard, Pal *et al*. proposed a methodology which combined the terms generated from WordNet and two local QE (23) term selection techniques [i.e. KLD (24) and RSV (25)]. They showed that precision of retrieval model could be improved by extending the query with candidate terms generated from local and global QE (26). Abdulla *et al*. combined terms from both global and local QE. For global QE, they used knowledge resources like PubMed (27) and MetaMap (28), whereas for local QE, Lavrenko relevance feedback (LRF) (29) and MFT (30) techniques were used. A linear combination approach was introduced to combine the scores generated by individual techniques. This combined score was used to select the final QE terms. They selected one method from global QE and one from local QE. By doing so, they experimented with various combination pairs and found that the best performance was obtained using linear combination approach on PubMed (https://www.ncbi.nlm.nih.gov/pubmed/) and LRF (22).

In our experimentation, we have exploited pseudo relevance feedback in which documents are ranked against particular user query. Top ranked k documents are selected as relevant for the selection of candidate of expansion terms. As there are no explicit defined criteria to select threshold (top k) for documents, there is a strong chance that arbitrarily selected threshold may cause document misclassification problem as some known relevant documents may get wrongly classified as relevant and vice versa. Traditionally used chi-square does not tackle mentioned problem while selecting expansion terms. We proposed a modified version of ‘Chi-Square’ which is able to alleviate the problem of document misclassification occurred due to selection of arbitrary threshold. We have evaluated our proposed term selection algorithm against eight state-of-the-art term selection algorithms and have shown the overall comparison. We have also tested the effect of stemming on information retrieval in particularly biomedical domain.

## Methodology

**Figure 1.**
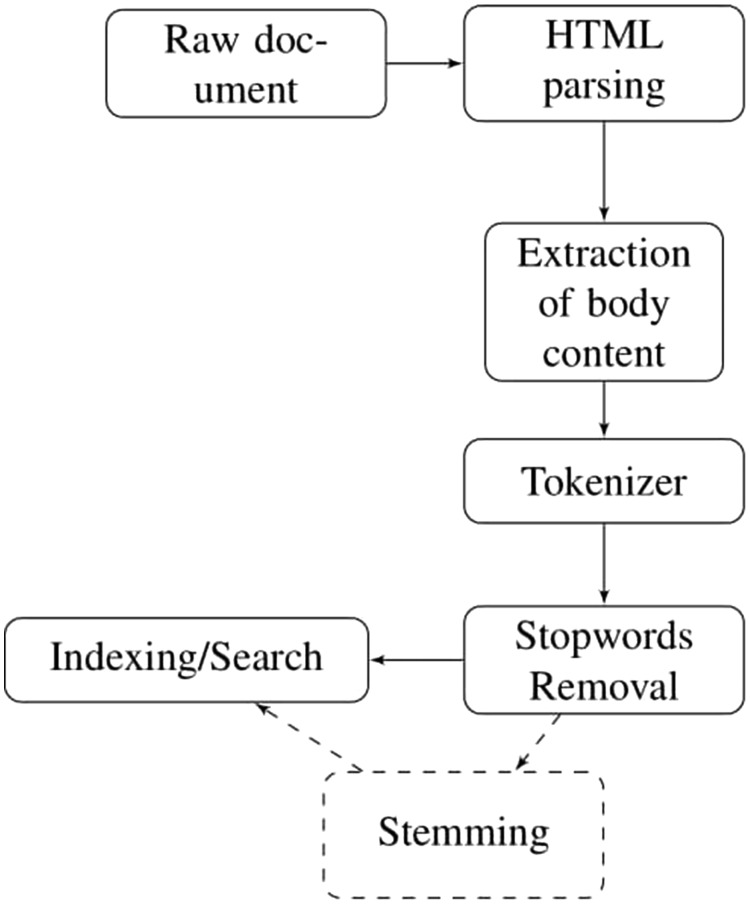
Pre-processing.

This section presents the methodology of pseudo relevance feedback emphasizing on the pre-processing of dataset. The dataset obtained from TREC website exists in HTML format having irrelevant information like email addresses, article digital signature, journal publishing dates and years etc. In order to remove this irrelevant content from the dataset, Apache Tika parser (https://tika.apache.org/0.7/parser.html) is used. Furthermore, all stop words such as is, am, are, about, etc. are removed from the dataset and user query by exploiting the default stop words list of solr named as ‘stop.txt’. It contains 33 English stop words. After this, we converted all the terms into their base form using Porter Stemmer. The steps involved in pre-processing of HTML documents are shown in Figure 1.

To measure the effect of stemming on the performance of retrieval task, we have indexed the dataset with and without stemming.

Performance of pseudo relevance feedback depends upon two significant factors: number of top relevant documents retrieved by document retrieval model, and term selection algorithm (20). Famous documents retrieval models are Okapi BM25, language models [unigram, bi-grams, n-grams (23)], TF-IDF etc. In our experimentation, we have used Okapi BM25 as our document retrieval model.

**Figure 2.**
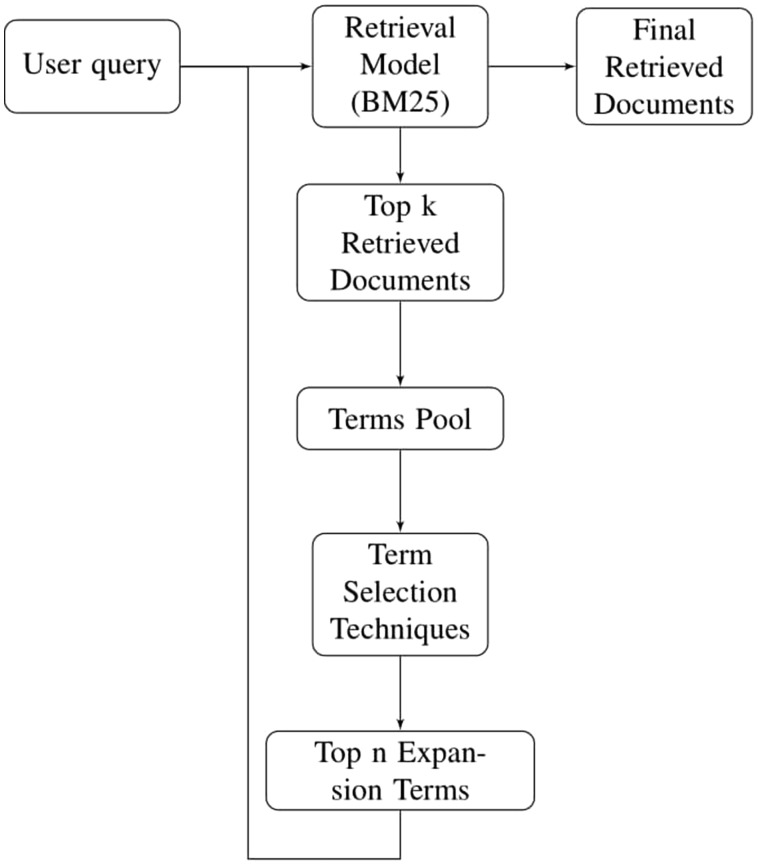
Methodology for pseudo relevance feedback.

Before feeding the user query to document retrieval model, all stop words are removed from user query. Since we have two different types of datasets i.e. stemmed and non-stemmed, therefore, user query is stemmed only for stemmed dataset. User query is then provided to document retrieval model which retrieves a list of ranked documents. Top k ranked documents are chosen for pseudo relevance feedback and only unique terms of these documents are used to create term pool. Various term selection techniques (mentioned in Section 5) are used to rank the terms for QE. Only top *n* terms are used to expand particular user query which is then sent back to retrieval model for final document retrieval. Using this expanded query, final ranked documents are retrieved. Figure 2 illustrates all the phases of PRF technique sequentially.

### Okapi bm25 weighting algorithm

Okapi BM25 is a probabilistic model that not only assigns weights to documents but also rank them according to their relevance against particular query. It has been widely used in biomedical domain for retrieval of information. Mathematical expression of document ranking is given as (31):

(1) $$weight=SJ\;.\;\frac{\left(k_{1}+1\right).{freq}_{id}}{K1.\left[\left(1-b\right)+b.\left(\frac{dl}{avdl}\right)\right]+{freq}_{id}}\times \;\frac{\left(k_{3}+1\right).{freq}_{iq}}{k_{3}+{freq}_{k3+{freq}_{iq}}\;}$$

where

- *k*1 and *k*_3_ are the parameters that are used to weight the effect of term frequency in document and query, whereas *b* is used as tuning constant to control normalization.
- *freq_id_* depicts the frequency of the occurrence of the term in document *d*.
- *freq_iq_* is the occurrence frequency of term in query *q*.
- *dl* and *avdl* illustrate document length and average document length in the corpus, respectively.

whereas,

*SJ* is the Robertson Sparck Jones weight, calculated using the formula below

(2) $$SJ=\log\frac{\left(rt+0.5)\;/(\left|R\right|-rt+0.5\right)}{\left(n-rt+0.5)\;/(N-n-\left|R\right|+0.5\right)}$$

where |*R*| is the number of relevant documents of a specific topic, *rt* is the number of relevant documents that contain the term *i*, *N* is the total documents present in the corpus and *n* denotes the number of documents containing that term.

### Term selection metrics

It is pretty obvious that corpus may have redundant and irrelevant terms that can cause query drift. To avoid this, all terms of corpus are ranked on the basis of statistical information used in various term ranking methods. In this section we will discuss eight such term ranking methods in context of QE.

### A. Kullback–Leibler divergence (KLD)

KLD (24) is widely used technique in information theory (32), statistical language modeling based speech processing and natural language applications (25). It assigns score to terms based on their probability in relevant documents and corpus.

(3) $$KLD\left(term\right)=P_{R}\left(term\right)\log\frac{P_{R}(term)}{P_{C}(term)}$$

where *P_R_*(*term*) is the probability of term’s presence in top retrieved relevant documents *R*. It can be calculated as:

(4) $$P_{R}\left(term\right)=\frac{\sum_{D\epsilon R}tf\left(term||D\right)}{\sum_{D\epsilon R}\sum_{term\;\epsilon \;D}tf\left(term||D\right)}.$$

And *P_C_*(*term*) is the probability of term’s presence in the corpus, calculated as:

(5) $$P_{C}\left(term\right)=\frac{\sum_{D\epsilon C}tf\left(term||D\right)}{\sum_{D\epsilon C}\sum_{term\;\epsilon \;D}tf\left(term||D\right)}.$$

Equation (3) is used to assign scores to terms present in the term pool. This technique assigns scores fall in the range of 0–1. The term having 0 score is considered as irrelevant term. Similarly, a score of 1 shows that the term is an excellent candidate for QE.

### B. Co-occurrence based query expansion

Co-occurrence is a term association based method used to assign scores to the terms present in the term pool. This method assigns score by measuring the relationship of candidate terms with query words (32). Rijsbergen (33) has described it as an algorithm that finds relationship between corpus and query terms. In order to find the co-occurrence association between two terms, co-efficients like CoJaccard, CoDice and Cosine are used. It can be calculated as:

(6) $$CoDice({term}_{i},{term}_{j})=\frac{{df}_{ij}}{{df}_{i}+\;{df}_{i}-{df}_{ij}}$$

where *df_i_* and *df_j_* are the frequency of documents in which term *i* and term *j* occur, respectively. Similarly, *df_ij_* is the number of documents in which both terms *i* and *j* occur together.

Expanding the query with highly similar terms may also cause query drift problem. In order to avoid query drift, the concept of inverse document frequency (IDF) is used. To handle this problem, codegree is calculated which also caters IDF as well. Let *qi* be the query term and *ct* be the candidate term, then codegree and IDF can be calculated using following expression

(7) $$Codegree\left(q_{i},ct\right)={\text{log}}_{10}\left(CoDice\left(q_{i},ct\right)+1\right).(\frac{IDF\left(ct\right)}{\;{\text{log}}_{10}\left(D\right)}).\;$$

And

(8) $$IDF\left(ct\right)={\text{log}}_{10}(\frac{N}{N_{c}})$$

where *N_c_* is the number of documents in corpus that have candidate term *ct*, *N* is the total number of documents present in corpus and *D* is the number of top retrieved documents. To obtain the value for a candidate term against all query terms, following formula can be used:

(9) $${Cooccurrence}_{final}\left(Q,ct\right)=\prod_{q_{i\;\epsilon \;Q}}\left(Codegree\left(q_{i},ct\right)\right).$$

### C. Information gain (IG)

IG is an algorithm that utilizes the knowledge about the presence or absence of particular term in documents to find the degree of class prediction (34). Let $C=\{C_{1},C_{2}\}$ be the set of classes where C1 belongs to top retrieved relevant documents and C2 belongs to non-relevant documents.

Value of IG for term *t* can be calculated as:

(10) $$IG\left(t\right)=-\sum_{j=1}^{\left|C\right|}P\left(c_{j}\right)\;\text{log}\;P\left(c_{j}\right)+P\left(t\right)\sum_{j=1}^{\left|C\right|}P\left(C_{j}||t\right)\;\;\text{log}\;P\left(C_{j}||t\right)\;+P(\overset{-}{t})\sum_{j=1}^{\left|C\right|}P\left(C_{j}|\overset{-}{|t}\right)\log P\left(C_{j}|\overset{-}{|t}\right)$$

where P (t) is the probability of term *t*’s occurrence, $\overset{-}{t}$ denotes non-occurrence probability i.e. $P\left(\overset{-}{t}\right)=1-P(t)$. $P\;(c_{j}|t)$ is the conditional probability that the *j*^th^ class occurs given term *t*. Similarly, $P(c_{j}|t)$ stands for the conditional probability of *j*^th^ class given the term *t* is non-existent, whereas $P(c_{j})$ is the probability of *j*^th^ class itself. This value is used to measure the importance of a term with respect to the two classes. This gives the score to the terms present in term pool. Ultimately high scoring terms can then be used for QE purpose.

### D. Probabilistic relevance feedback (PRF)

This measure assigns score to the terms present in term pool by calculating their probability in relevant and non-relevant documents (35). A term having higher probability in relevant class is considered more suitable candidate term for QE. Mathematical expression of PRF is obtained as:

(11) $$PRF\left(t\right)=\frac{P_{relevance}(term)}{P_{non-relevance}(term)}$$

where $P_{relevance}(term)$ is the probability of term in relevant documents and $P_{non-relevance}(term)$ is the probability of term in non-relevant documents.

### E. Chi-square (CS)

A statistical measure used to measure the divergence of two events is known as chi-square (36). For a term *t*, it measures how much independent *t* is from relevant and irrelevant class. The lesser the independence, the higher will be the score for that term. Mathematical expression of chi-square is given below

(12) $$Chi-Square=\frac{{\left[p_{R}\left(t\right)-p_{C}\left(t\right)\right]}^{2}}{p_{C}\left(t\right)}$$

where $p_{R}(t)$ is the probability of term *t* present in relevant documents, and $p_{C}(t)$ is the probability of term in corpus. In experimentation we also used chi-square version without square used by (11).

### F. Lavrenko relevance feedback (LRF)

This technique uses the formula derived from Lavrenko relevance model (37). It is the technique based on language model. The score for the QE terms can be found by using the formula:

(13) $$Score\left(t\right)=\sum_{all\;R}\;\text{log}\;\frac{P(t|M_{R})}{P(t|G)}.\;$$

In above equation, P(t|G) is the probability of occurrence of the term t in collection. Whereas, $P(t|M_{R})$ can be found using the formula below:

(14) $$P\left(t|{|M}_{R}\right)=\lambda \;\times \frac{TF\left(t,R\right)}{\sum_{t\;\epsilon \;R}TF\left(t,R\right)}+(1-\lambda )\times P(t|G)$$

where TF(t,R) is the frequency of the term in relevant document *R* and the denominator is the summation of all the term frequencies for a relevant document. The *λ* is the parameter that can be adjusted during experimentation. Researchers have found that *λ*=0.6 shows best results (22).

### Proposed term selection metric: document frequency chi-square (DFC)

Chi-square is one of the widely used algorithms for term selection in text classification. It has been used by Carpineto *et al*. for pseudo relevance feedback based term selection but unfortunately its performance was not up to the mark because term selection for QE in pseudo relevance feedback is very different from term selection in text classification. In pseudo relevance feedback, there exist only two classes which are highly skewed. We first retrieve documents based on user query and select top k documents as relevant while the rest of the documents are treated as non-relevant. However, there is no defined criterion to choose the threshold between relevant and non-relevant ranked list of documents. There is a possibility that a non-relevant document may get classified as relevant document. Similarly, possibility of getting a relevant document in non-relevant class also exists. In order to fully understand the effect of this thresholding, let us consider a corpus of 10 documents which contain three documents (D1,D2,D3) of actual relevant class and rest are from non-relevant class. In pseudo relevance feedback, after document ranking, if we decide threshold at D4, we will get the following sets of documents:

R={D1,D2,D3,D4}

NR={D5,D6,D7,D8,DD,D10}

Let there be terms *t*_1_–*t*_50_ in corpus. We consider a scenario in which *t*_1_ occurs 10 times in *R* however it is only occurring in *D*4 document. The same term occurs three times in *NR*, such that it appears two times in *D*5 and one times in *D*6 document. When distribution based on term frequency is considered, chi-square will consider *t*_1_ as a good term for QE which is not true. Now if the distribution is considered in context of document frequency which is binary in nature and only considers the presence of term in documents, we notice that using this distribution, document frequency of *t*_1_ is only 1 in *R* whereas it is 2 in *NR*. As *t*_1_ has higher document frequency in non-relevant class, therefore DFC will not rank it as a discriminative term. DFC not only considers the term presence in relevant documents, but also keeps track of other important factors like terms’ absence in relevant class and similarly term presence and absence in non-relevant class as well. Mathematically, its formula can be written as:

(15) $$DFC\;=\frac{{\left({tdf}_{r}-{tdf}_{c}\times {ratio}_{r}\right)}^{2}}{{tdf}_{c}\times {ratio}_{r}}+\frac{{\left(\bar{{tdf}_{r}}-\bar{{tdf}_{c}}\times {ratio}_{r}\right)}^{2}}{\bar{{tdf}_{c}}\times {ratio}_{r}}+\frac{{\left({tdf}_{nr}-{tdf}_{c}\times {ratio}_{nr}\right)}^{2}}{{tdf}_{c}\times {ratio}_{nr}}+\frac{{\left(\bar{{tdf}_{nr}}-\bar{{tdf}_{c}}\times {ratio}_{nr}\right)}^{2}}{\bar{{tdf}_{c}}\times {ratio}_{nr}}$$

such that

(16) $${ratio}_{r}=\frac{size\;of\;relevant\;class}{corpus\;size}$$

(17) $${ratio}_{nr}=\frac{size\;of\;nonrelevant\;class}{corpus\;size}$$

where

${tdf}_{r}$= term document frequency in relevant class,

${tdf}_{c}$= term document frequency in corpus,

${tdf}_{nr}$= term document frequency in non-relevant class,

$\bar{{tdf}_{nr}}$= term absence in non-relevant class,

$\bar{{tdf}_{r}}$= term absence in relevant class and

$\bar{{tdf}_{c}}$= term absence in corpus.

### Dataset and evaluation measure

In order to address the information retrieval system that targets the needs of biomedical scientists and geneticists, TREC 2006 Genomic Track (38) dataset is selected. This dataset consists of 162 259 documents having total 1 437 356 250 unique terms from 49 journals published electronically at Highwire Press. These are HTML documents obtained by using web crawler on the Highwire Press website. The full collection is 12.3 GB in size.

MAP is used to evaluate the performance of nine term selection algorithms using Okapi BM25 as retrieval model. This evaluation measure is widely used in information retrieval system. Mathematical expressions of average and MAP are given below

#### a: Average precision

This measure compares the documents ranked by retrieval model with pre-defined set of documents ranked by domain experts against particular query.

(18) $$AverageP=\frac{\sum_{r=1}^{N}\left(P(r)\times rel(r)\right)}{C_{rt}}\;$$

where

***r*** is rank,

*N* denotes the number of retrieved documents,

rel(r) is a function that tells whether a document is relevant or not (binary) and

P(r) stands for precision.

#### b: Mean average precision

It summarizes the ranking results obtained from multiple queries by averaging the AverageP.

(19) $$MAP=\frac{\sum_{q=1}^{Q}AverageP(q)}{\left|Q\right|}.\;$$

### Practical illustration of TREC data

This section summarizes the background of strategical decisions taken in context of typical behavior of the system over different queries. It also depicts the source of query drift in quest of further improvements while producing and comparing results.

Table 1 shows performance difference of two algorithms (DFC and chi-square) and baselines for 36 queries of TREC Dataset. All results have been calculated on the following benchmark: documents =40, top terms =10. Delta(DFC-CS) shows the difference in precision of DFC and Chi-Square. The most positive value of delta(DFC-CS) shows that DFC has outperformed chi-square. On the other hand, the most negative value depicts victory of chi-square over DFC with a huge margin. By observing the differences, we notice that query 201 has the most positive value of delta(DFC-CS) whereas query 207 has most negative.

**Table 1.** Summary of difference in precision for 36 TREC queries

Queries	Precision		Base line	PΔ (Term selection—base line)		
	DFC	chi-square		Δ (DFC-CS)	Δ (DFC-BS)	Δ (CS-BS)
200	0.3819	0.4123	0.3796	−0.0304	0.0023	0.0327
201	0.9755	0.5599	0.5825	0.4156	0.393	−0.0226
202	0.039	0.0439	0.0528	−0.0049	−0.0138	−0.0089
203	0.6378	0.6334	0.6393	0.0044	−0.0014	−0.0059
204	0.6649	0.6637	0.6423	0.0012	0.0226	0.0214
205	0.1658	0.1349	0.1954	0.0309	−0.0296	−0.0605
206	0.5315	0.3412	0.4337	0.1903	0.0978	−0.0925
207	0.0744	0.1364	0.0661	−0.062	0.0083	0.0703
208	0.4222	0.462	0.264	−0.0398	0.1583	0.1981
209	0.5115	0.1994	0.2644	0.3121	0.2471	−0.065
210	0.0651	0.0737	0.0764	−0.0086	−0.0113	−0.0027
211	0.4594	0.2348	0.3481	0.2246	0.1113	−0.1133
212	0.3631	0.3555	0.3941	0.0076	−.031	−0.0386
213	0.5186	0.5587	0.5306	−0.0401	−.0121	0.0281
214	0.558	0.4891	0.5408	0.0689	0.0171	−0.0517
215	0.39	0.3247	0.4557	0.0653	−0.0657	−0.131
216	0.1085	0.0849	0.0808	0.0236	0.0277	0.0041
217	0.0031	0.001	0.0051	0.0021	−0.002	−0.0041
218	0.2355	0.2021	0.2834	0.0334	−0.0479	−0.0813
219	0.0201	0.0875	0.0839	−0.0619	−0.0638	0.0036
220	0.9151	0.9242	0.8556	−0.0092	0.0595	0.0687
221	0.0001	0.0001	0.0001	0.0001	0.0001	0.0001
222	0.0964	0.0648	0.0852	0.0316	0.0112	−0.0204
223	0.2323	0.1166	0.4105	0.1157	−0.1782	−0.2939
224	0.0298	0.0215	0.2268	0.0083	−0.197	−0.2053
225	0.0909	0.0833	0.0164	0.0076	0.0745	0.0669
226	0.7449	0.6847	0.303	0.0603	0.4419	0.3817
227	0.1663	0.1455	0.1716	0.0208	−0.0053	−0.026
228	0.005	0.0046	0.005	0.0004	0	−0.0004
229	0.6221	0.4988	0.5042	0.1232	0.1179	−0.0053
230	0.1966	0.0864	0.0905	0.1102	0.1061	−0.0041
231	0.1169	0.038	0.1344	0.079	−0.0175	−0.0964
232	0.0841	0.0797	0.0833	0.0044	0.0008	−0.0036
233	0.1	0.0488	0.0875	0.0513	0.0126	−0.0387
234	0.1158	0.085	0.1162	0.0307	−0.0004	−0.0311
235	0.1298	0.1315	0.1771	−0.0016	−0.0473	−0.0457
**MAP**	**0.2992**	**0.2504**	**0.2663**	**0.0489**	**0.0329**	**−0.0159**

Delta(DFC-BS) and delta(CS-BS) are the differences in the performance of information retrieval system after applying QE using algorithms (DFC and chi-square) and without applying any QE (baseline).

These columns show the effect on the performance after applying QE techniques. Positive value of the delta shows an increase in performance after applying QE whereas negative value depicts decline in performance due to QE. It is pretty easy to see that negative value of delta in both cases is directly proportional to the query drift. It can be seen from the table that 16 out of 35 queries have shown a decrease in performance due to query drift using DFC. On the other hand, by applying QE using chi-square, only 9 out of 36 queries have shown an improved performance. For delta(DFC-BS), the best performance has observed for query 225 and for delta (CS-BS), query 226 has marked the most increase in precision after applying QE. Highlighted values at the bottom of the table illustrates mean average precision difference of mentioned algorithms.

In order to further explore chi-square term selection algorithms, query 201 and 207 are selected as they have revealed best performance for DFC and chi-square, respectively. These two algorithms are applied again on query 201 and 207 to obtain top 10 terms from top 40 retrieved documents. The selected terms are listed in Tables 2 and 3.

**Table 2.** Top 10 terms selected by chi-square and DFC for query 201 (precision is shown by adding each term to query)

Top 10 terms ranked by DFC											
Terms	Baseline	calipel	v599e	nature00766	mouriaux	v600e	trovisco	braf	418934a	shieldsj	klintenas
Precision	0.5825	0.6426	0.9320	0.9569	0.8647	0.9267	0.9371	0.9404	0.9676	0.9676	0.9735
Top 10 terms ranked by chi-square											
Terms	Baseline	braf	ras	raf	transgelin	cref	9nc	vmm12	v600e	uveal	kras
Precision	0.5825	0.7632	0.7960	0.8127	0.6528	0.5598	0.5018	0.5657	0.7561	0.7977	0.8307
Query Terms	genes	associated	cancer

**Table 3.** Top 10 terms selected by chi-square and DFC for query 207 (precision is shown by adding each term to query)

Top 10 terms ranked by DFC											
Terms	Baseline	etidronate	alendronate	bisphosphonates	didronel	risedronate	ibandronate	bisphosphonate	art271	int1999; 9	pprice
Precision	0.06613926	0.0798	0.0946	0.147	0.1557	0.1523	0.1777	0.1874	0.1736	0.1689	0.164
Top 10 terms ranked by chi-square											
Terms	Baseline	etidronate	fetuin	bisphosphonates	incadronate	paget’s	pamidronate	aminobisphosphonates	ibandronate	bisphosphonate	tiludronate
Precision	0.06613926	0.0798	0.0695	0.1265	0.1604	0.1691	0.2286	0.204	0.2056	0.2073	0.2609
Query Terms	toxicities	associated	etidronate								

Original query is expanded by adding one term at a time and precision is measured just to reveal the positive or negative effects of newly added term over QE. Results of incremental QE obtained after iterating over all 10 terms are shown in Tables 2 and 3. As observed from the tables, expanding user query with selected terms has marked a reasonable boost in the performance of specified query.

Tables 4 and 5 depict the unique terms selected by chi-square and DFC for queries 201 and 207, respectively. Both tables also show the document frequency based parameters $\left({tdf}_{r},\;\bar{{tdf}_{r}},\;{tdf}_{nr},\;\bar{{tdf}_{nr}}\right)$ as well as the probabilities used by chi-square. To lay out a clear picture of the importance of terms against each algorithm, ranks of these unique terms as determined by their scores of chi-square and DFC are also shown.

**Table 4.** Scores of 18 unique terms selected by chi*-*square and DFC for query 201 on 40 documents

Terms	*P* (relevance)	*P* (corpus)	${tdf}_{r}$	$\bar{{tdf}_{nr}}$	${tdf}_{nr}$	$\bar{{tdf}_{r}}$	CS	${Rank}_{CS}$	DFC	${Rank}_{DFC}$
braf	0.00082	3.39E-07	14	162150	69	26	1.9978	1	9558.411	7
ras	0.01085	9.00E-05	38	149277	12942	2	1.2877	2	411.485	16
raf	0.00652	3.36E-05	40	157245	4974	0	1.2516	3	1254.757	13
transgelin	0.00035	1.26E-07	1	162197	22	39	0.95	4	174.442	18
cref	0.00037	1.64E-07	1	162203	16	39	0.8312	5	236.704	17
9nc	0.00015	4.31E-08	1	162216	3	39	0.5542	6	1012.394	14
vmm12	0.00012	3.27E-08	1	162219	0	39	0.4639	7	4055.5	11
v600e	0.00012	3.34E-08	5	162217	2	35	0.4351	8	14481.607	4
uveal	0.00031	2.44E-07	3	162144	75	37	0.3911	9	462.41	15
kras	0.00043	4.81E-07	6	162130	89	34	0.3783	10	1526.484	12
calipel	0.00002	5.57E-09	5	162219	0	35	0.079	12	20278	1
v599e	0.00011	4.80E-08	8	162214	5	32	0.2523	11	19960.861	2
nature00766	0.00003	2.23E-08	12	162199	20	28	0.0444	15	18238.237	3
mouriaux	0.00003	9.04E-09	5	162217	2	35	0.0759	13	14481.607	5
trovisco	0.00001	3.48E-09	3	162219	0	37	0.0494	14	12166.65	6
418934a	0.00001	7.65E-09	5	162213	6	35	0.0224	17	9212.159	8
shieldsj	0.00001	2.09E-09	2	162219	0	38	0.0296	16	8111.05	9
klintenas	0.00001	1.39E-09	2	162219	0	38	0.0197	18	8111.05	10

**Table 5.** Scores of 18 unique terms selected by Chi*-*square and DFC for query 207 on 40 documents

Terms	*P* (relevance)	*P* (corpus)	${tdf}_{r}$	$\bar{{tdf}_{nr}}$	${tdf}_{nr}$	$\bar{{tdf}_{r}}$	CS	${Rank}_{CS}$	DFC	${Rank}_{DFC}$
etidronate	0.0019	3.10E-07	40	162186	33	0	11.64	1	88891	1
fetuin	0.0029	1.70E-06	4	161944	275	36	4.941	2	225.14	16
bisphosphonates	0.0017	9.03E-07	32	161948	271	8	3.208	3	13674	3
incadronate	0.0003	5.90E-08	4	162214	5	36	1.525	4	7205.69	12
paget’s	0.0008	3.70E-07	6	162119	100	34	1.728	5	1366.93	15
pamidronate	0.0012	7.50E-07	18	162081	138	22	1.918	6	8399.17	11
aminobisphosphonates	0.0003	7.60E-08	6	162202	17	34	1.184	7	6339.73	13
ibandronate	0.0006	3.60E-07	12	162184	35	28	0.999	8	12411	6
bisphosphonate	0.0007	4.80E-07	26	162023	196	14	1.019	9	12320.1	7
tiludronate	0.0001	3.40E-08	5	162206	13	35	0.294	10	5626.01	14
alendronate	0.0005	6.40E-07	24	162096	123	16	0.39	12	15865.1	2
didronel	1.80E-05	5.50E-09	4	162218	1	36	0.059	16	12976.3	4
risedronate	0.0003	1.90E-07	12	162186	33	28	0.473	11	12963.5	5
art271	1.40E-05	2.08E-09	3	162219	0	37	0.098	14	12166.7	8
int1999; 9	1.40E-05	2.08E-09	3	162219	0	37	0.098	15	12166.7	9
pprice	3.20E-05	7.60E-09	4	162217	2	36	0.135	13	10812.3	10

As shown in the Table 4, chi-square has assigned highest score to the term braf while DFC ranks calipel as the top term. A close inspection of document frequency parameters show that braf is present in 14 relevant documents and 69 non-relevant documents. On the other hand, calipel is present in five documents of relevant class and it is entirely absent in non-relevant documents. Due to this reason, DFC considers it a highly discriminative term to differentiate between relevant and non-relevant class. Similarly, we observe second term ranked by both algorithms. DFC has placed v5899e at second rank whereas is selected as second best term by chi-square. We explain this by observing the fact that is present more times in non-relevant documents as compared to v599e.

Likewise, other terms can also be observed from the table. Similarly, from Table 5 it can be seen that etidronate is ranked as the best term by both algorithms. DFC has selected alendronate as second best term and chi-square placed fetuin at second rank. Fetuin is present in only 4 documents of relevant class and 275 of non-relevant class documents. However, alendronate is present in 24 relevant documents and 123 non-relevant documents. By analyzing and comparing these parameters, it is pretty easy to see that alendronate is more suitable candidate than fetuin as it is present more times in relevant documents and also has lesser occurrence in non-relevant class.

### Experimental setup and results

We use an open source search platform known as ‘Solr’ (39) for experimentation. It includes features of full text search and real time indexing. In experimentation, Okapi BM25 is used as retrieval model. In this section we briefly explain about experimental setup and compare the results of all term selection techniques against defined test points.

#### A. Results without stemming

To analyze the effect of pre-processing on biomedical data, we have used two different methods for indexing of the corpus documents as discussed in the Section 3. This section depicts the results of nine term selection algorithms in the form of tables at pre-defined test points. Expectedly, all feature selection techniques do not produce their peak results at the same defined set of parameters. These parameters are number of top retrieved relevant documents and candidate expansion terms that get merged with the query. For sake of laying out the clear picture of the performance of pseudo relevance feedback and better comparison of term ranking algorithms, we have shown a graph containing the peak results only against the best parameters of terms for all techniques found from below mentioned tables.

Tables 6–10 illustrate MAP of nine term selection algorithms on pre-defined benchmark test points at top terms (5, 10, 15, …, 50) and documents (10, 20, …, 50). Boldface values in these tables indicate the highest performance of a particular term selection algorithm across all the mentioned term selection algorithms at a specific number of terms.

**Table 6.** Mean average precision of nine term selection algorithms by choosing digit 10 as threshold to divide ranked corpus documents as relevant and non-relevant

No. of terms	FR metrics								
	Chi-square	Chi	DFC	KLD	RSV	CoDice	IG	LRF	PRF
5	0.2661	0.2632	0.2748	0.2724	0.2713	0.2696	0.2712	**0.2859**	0.2661
10	0.2547	0.2649	0.2801	0.2665	0.2619	0.2777	0.2801	**0.2884**	0.2692
15	0.2506	0.2617	0.281	0.2539	0.2647	0.2821	0.2817	**0.2839**	0.2653
20	0.2569	0.2624	0.2815	0.2543	0.2493	0.272	0.2798	**0.2842**	0.2643
25	0.2619	**0.2587**	0.2814	0.258	0.2439	0.2726	0.279	0.279	0.2616
30	0.2593	**0.259**	0.28	0.2501	0.2456	0.2697	0.279	0.2746	0.2603
35	0.2627	**0.2591**	0.28	0.2488	0.2488	0.2675	0.2717	0.2701	0.2603
40	0.2596	**0.255**	0.278	0.2499	0.2466	0.2648	0.2678	0.2666	0.2577
45	0.2606	**0.257**	0.2764	0.2455	0.25	0.2647	0.2652	0.2644	0.2558
50	0.2595	**0.2576**	0.2765	0.2445	0.2426	0.2654	0.2596	0.2648	0.2565

**Table 7.** Mean average precision of nine term selection algorithms by choosing digit 20 as threshold to categories ranked corpus documents as relevant and non-relevant

No. of terms	FR metrics								
	Chi-square	Chi	DFC	KLD	RSV	CoDice	IG	LRF	PRF
5	0.2574	0.2614	0.2859	**0.2925**	0.2824	0.2727	0.2700	0.2835	0.2624
10	0.2668	0.2519	**0.2892**	0.2754	0.2657	0.2777	0.2666	0.2787	0.2651
15	0.2660	0.2531	**0.2878**	0.2600	0.2553	0.2714	0.2674	0.2660	0.2637
20	0.2616	0.2515	**0.2890**	0.2556	0.2495	0.2672	0.2677	0.2605	0.2663
25	0.2656	0.2514	**0.2892**	0.2522	0.2441	0.2657	0.2663	0.2587	0.2643
30	0.2745	0.2504	**0.2910**	0.2443	0.2381	0.2667	0.2635	0.2554	0.2591
35	0.2642	0.2496	**0.2887**	0.2406	0.2375	0.2620	0.2608	0.2530	0.2619
40	0.2613	0.2482	**0.2912**	0.2391	0.2381	0.2598	0.2580	0.2495	0.2598
45	0.2630	0.2452	**0.2891**	0.2358	0.2380	0.2601	0.2598	0.2476	0.2577
50	0.2630	0.2460	**0.2903**	0.2343	0.2338	0.2586	0.2569	0.2441	0.2565

**Table 8.** Mean average precision of nine term selection algorithms by choosing digit 30 as threshold to divide ranked corpus documents as relevant and non-relevant

No. of terms	FR metrics								
	Chi-square	Chi	DFC	KLD	RSV	CoDice	IG	LRF	PRF
5	0.2504	0.2372	0.2925	**0.2954**	0.2758	0.2748	0.2798	0.2856	0.2559
10	0.2617	0.2298	**0.2951**	0.2720	0.2569	0.2642	0.2674	0.2760	0.2527
15	0.2594	0.2294	**0.2935**	0.2657	0.2639	0.2591	0.2628	0.2580	0.2521
20	0.2574	0.2290	**0.2885**	0.2605	0.2544	0.2612	0.2586	0.2577	0.2523
25	0.2597	0.2299	**0.2894**	0.2554	0.2453	0.2644	0.2507	0.2570	0.2552
30	0.2619	0.2278	**0.2851**	0.2513	0.2402	0.2648	0.2502	0.2552	0.2531
35	0.2575	0.2280	**0.2852**	0.2507	0.2356	0.2628	0.2486	0.2499	0.2521
40	0.2531	0.2260	**0.2828**	0.2337	0.2300	0.2599	0.2470	0.2431	0.2490

**Table 9.** Mean average precision of nine term selection algorithms by choosing digit 40 as threshold to categories ranked corpus documents as relevant and non-relevant

No. of terms	FR metrics								
	Chi-square	Chi	DFC	KLD	RSV	CoDice	IG	LRF	PRF
5	0.2480	0.2508	**0.2897**	0.2744	0.2674	0.2636	0.2699	0.2705	0.2523
10	0.2533	0.2369	**0.2983**	0.2702	0.2599	0.2597	0.2586	0.2595	0.2525
15	0.2616	0.2381	**0.2983**	0.2659	0.2578	0.2541	0.2514	0.2427	0.2533
20	0.2613	0.2293	**0.2926**	0.2611	0.2447	0.2503	0.2473	0.2281	0.2523
25	0.2624	0.2249	**0.2862**	0.2490	0.2381	0.2512	0.2417	0.2425	0.2503
30	0.2599	0.2262	**0.2850**	0.2392	0.2316	0.2543	0.2403	0.2339	0.2481
35	0.2588	0.2216	**0.2880**	0.2326	0.2318	0.2532	0.2404	0.2276	0.2415
40	0.2541	0.2190	**0.2866**	0.2362	0.2338	0.2538	0.2432	0.2223	0.2413
45	0.2518	0.2208	**0.2844**	0.2374	0.2330	0.2517	0.2423	0.2304	0.2389
50	0.2503	0.2195	**0.2836**	0.2365	0.2256	0.2499	0.2421	0.2284	0.2349

**Table 10.** Mean average precision of nine term selection algorithms by choosing digit 50 as threshold to classified ranked corpus documents as relevant and non-relevant

No. ofterms	FR metrics								
	Chi-square	Chi	DFC	KLD	RSV	CoDice	IG	LRF	PRF
5	0.2491	0.2342	**0.2926**	0.2810	0.2630	0.2502	0.240	0.2479	0.2540
10	0.2423	0.233	**0.2987**	0.2575	0.2517	0.2489	0.2276	0.2485	0.2530
15	0.2629	0.2212	**0.3015**	0.2579	0.2539	0.2441	0.2366	0.2400	0.2572
20	0.2685	0.2197	**0.2994**	0.2561	0.2403	0.2464	0.2397	0.2204	0.2552
25	0.2633	0.2192	**0.2904**	0.2459	0.2354	0.2450	0.2338	0.2134	0.2553
30	0.2613	0.2153	**0.2871**	0.2394	0.2264	0.2413	0.2324	0.2140	0.2517
35	0.2581	0.2137	**0.2898**	0.2366	0.2303	0.2426	0.2365	0.2088	0.2477
40	0.2566	0.2145	**0.2881**	0.2342	0.2211	0.2418	0.2289	0.1998	0.2447
45	0.2521	0.2132	**0.2875**	0.2304	0.2235	0.2408	0.2353	0.1982	0.2428
50	0.2509	0.2116	**0.2870**	0.2272	0.2176	0.2414	0.2303	0.1998	0.2413

Table 6 highlights the best performing term selection algorithms over following defined set of test points (i.e. top documents =10, top terms =5, 10, 15, 20, 25, 30, 35, 40, 45, 50). It can be clearly seen that LRF outperforms the rest of term selection algorithms at following test points *T *= 5, 10, 15, 20. Likewise, DFC exhibits best performance in the remaining test points. RSV does not perform up to the mark as its performance kept decreasing gradually with the increase in number of terms. KLD follow the footsteps of RSV but it somehow manages to beat RSV in a race of being called as worst performing algorithm.

It has also been observed that the performance of chi (without square) and PRF show an overall decline in score with gradual increase in number of top selected terms. We can also observe from the table that the scores of CoDice and IG kept increasing until the term test point *T* = 15, and for the remaining test points, decrease in performance is observed. On the other hand, chi-square follows a mix sort of trend as its performance kept decreasing slightly on couple of test points at first and then all of a sudden start increasing but then it gradually decreases for remaining term test points.

Table 7 illustrates the performance of term selection algorithms for 20 number of documents and top terms *T* = 5, 10, 15, 20, 25, 30, 35, 40, 45, 50. As the table suggests, it is pretty obvious to say that KLD outperforms the rest of term selection algorithms only at following test point *T* = 5. Surprisingly, DFC exhibits best performance in all the remaining test points. In addition, RSV does not perform up to the mark again even with the increase of top documents, as its performance (32) kept decreasing gradually with the increase in number of terms. The performance of IG, LRF and chi (without square) follow a pattern in which they have highest MAP at term test point =10, whereas for the rest of the test points, gradually decreasing scores are observed. Chi-square based on probability shows a peculiar behavior as the performance first arbitrarily increases with gradual increase of top selected terms. This increase in performance is observed until *T* = 30 and after that the performance drops and an almost constant score is observed. As far as CoDice and PRF are concerned, no clear pattern is observed in their performance. Some test points cause a slight increase or decrease in performance while others keep the performance constant.

In Table 8, we have depicted the results of term selection algorithms obtained at document test point =30 and for all defined terms test points =5, 10, 15, 20, 25, 30, 35, 40, 45, 50. As the table suggests, it is pretty obvious to say that KLD outperforms the rest of term selection algorithms only at following test point *T* = 5. Surprisingly, DFC exhibits best performance in all the remaining test points. In addition, Chi (without square) is the worst performer and its performance kept decreasing gradually with the increase in number of terms. LRF and IG start with a very good score at *T* = 5 but with the increase in number of top selected terms, their performance also kept getting worst. On the other hand, PRF follows an almost constant trend as the difference between its best and worst score is only 0.011. The performance of term selection algorithms such as chi-square, RSV and CoDice follow a mixed pattern. As the number of top terms are increased, the results of mentioned term selection algorithm sometimes increase and all of a sudden decrease at the very next test point.

For top document =40 and 50, we have shown the best performance of nine term selection algorithms in Tables 9 and 10, respectively. As the table suggests, it is pretty clear that DFC exhibits best performance in all the test points. Table 9 depicts that the performance of KLD, RSV, CoDice, IG and PRF keep decreasing gradually with the increase in number of terms. It also marks that Chi (without square) is the worst performer as it shows the least score at *T *= 50.

However, algorithms such as LRF and chi-square follow no clear pattern as their score vary from one test point to another by either decreasing or increasing suddenly.

While studying the performance of term selection algorithms in Table 10, we observe that LRF depicts the worse performance and shows gradual decrease in performance with increasing number of terms. KLD, RSV, CoDice and PRF also follow a decreasing pattern as they mark their best performance only at *T* = 5 and eventually kept getting decrease until term test point 50. Conversely, we observe that algorithms such as chi-square and IG show an unpredictable behavior in their performance. The scores of chi-square first increase up to *T* = 15, and then decrease as number of terms approaches to 50. IG shows an even more abrupt behavior as the score keeps on increasing and decreasing at different term test points.

**Figure 3.**
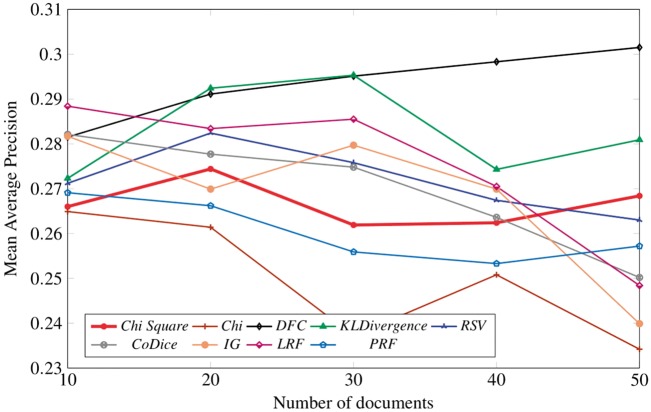
Peak results of nine term selection techniques.

Figure 3 result summarizes the performance of nine term selection algorithms in terms of MAP against number of documents. Trends of all term selection algorithms (chi-square, KLD, RSV, CoDice, IG, LRF) along with newly proposed technique (DFC) and baseline are shown only at peak values retrieved from Tables 6–10. As the graph suggests, it is pretty easy to see that DFC and KLD have outperformed the rest but in a straight comparison, DFC is a clear winner. Although at start there is a clear difference between the performance of DFC and LRF, but eventually with the increase in number of documents, DFC performance has gradually improved and reached the highest value of 0.3. As a result, we conclude that LRF outperforms the rest of the algorithms between 10 to nearly 15 documents, whereas the performance of DFC is highest for almost next 5 documents. For around next 10 documents, KLD has shown a slightly better performance than DFC but after that DFC has emerged as the winner among all term selection algorithms.

#### B. Results with stemming

This section compares the performance of the nine term selection algorithms before and after stemming.

Tables 11–15 depict the difference in MAP of nine term selection algorithms on the pre-defined test points (i.e. number of top documents =10, 20, 30, 40, 50 and number of top terms =5, 10, 15, …, 50). For every algorithm, this MAP difference is denoted by Delta and is calculated as:

(20) $$\Delta_{algorithm}={MAP}_{\left(before\;Stemming\right)}-{MAP}_{\left(after\;Stemming\right)}.\;$$

**Table 11.** Mean average precision difference of nine term selection algorithms at 10 documents

No. of terms	FR metrics								
	*^Δ^*Chi-square	*^Δ^*Chi	*^Δ^*DFC	*^Δ^*KLD	*^Δ^*RSV	*^Δ^*CoDice	*^Δ^*IG	*^Δ^*LRF	*^Δ^*PRF
5	0.0805	0.1032	0.0897	0.0155	0.1091	0.0784	0.0460	0.0625	0.0526
10	0.0850	0.1206	0.1124	0.0044	0.1107	0.0830	0.0557	0.0639	0.0458
15	0.0906	0.1399	0.1222	0.0164	0.1278	0.0948	0.0507	0.0628	0.0781
20	0.0898	0.1427	0.1105	0.0174	0.1289	0.0930	0.0544	0.0596	0.0674
25	0.1069	0.1318	0.1242	0.0194	0.1340	0.0980	0.0511	0.0608	0.0781
30	0.0901	0.1312	0.1247	0.0105	0.1273	0.0947	0.0545	0.0582	0.0818
35	0.0807	0.1294	0.1206	0.0203	0.1319	0.1012	0.0582	0.0572	0.0755
40	0.0870	0.1246	0.1185	0.0111	0.1358	0.0958	0.0649	0.0622	0.0798
45	0.0883	0.1286	0.1257	0.0161	0.1394	0.1007	0.0660	0.0646	0.0810
50	0.0816	0.1301	0.1242	0.0200	0.1434	0.0995	0.0658	0.0613	0.0806

**Table 12.** Mean average precision difference of nine term selection algorithms at 20 documents

No. ofterms	FR metrics								
	*^Δ^*Chi-square	*^Δ^*Chi	*^Δ^*DFC	*^Δ^*KLD	*^Δ^*RSV	*^Δ^*CoDice	*^Δ^*IG	*^Δ^*LRF	*^Δ^*PRF
5	0.0888	0.0832	0.0898	0.0367	0.1368	0.0679	0.0474	0.0510	0.0597
10	0.1042	0.1150	0.1050	0.0372	0.1529	0.0818	0.0607	0.0611	0.0820
15	0.1224	0.1356	0.1267	0.0372	0.1528	0.0871	0.0504	0.0713	0.0924
20	0.1267	0.1290	0.1369	0.0231	0.1438	0.0839	0.0555	0.0698	0.0864
25	0.1214	0.1321	0.1491	0.0236	0.1513	0.0813	0.0570	0.0713	0.0958
30	0.1259	0.1345	0.1545	0.0304	0.1429	0.0881	0.0644	0.0604	0.1046
35	0.1260	0.1353	0.1559	0.0268	0.1416	0.0891	0.0655	0.0591	0.1095
40	0.1217	0.1335	0.1621	0.0278	0.1400	0.0937	0.0656	0.0555	0.1077
45	0.1244	0.1315	0.1556	0.0309	0.1300	0.0930	0.0739	0.0551	0.1143
50	0.1218	0.1268	0.1609	0.0357	0.1220	0.0946	0.0775	0.0577	0.1165

**Table 13.** Mean average precision difference of nine term selection algorithms at 30 documents

No. ofterms	FR metrics								
	*^Δ^*Chi-square	*^Δ^*Chi	*^Δ^*DFC	*^Δ^*KLD	*^Δ^*RSV	*^Δ^*CoDice	*^Δ^*IG	*^Δ^*LRF	*^Δ^*PRF
5	0.0836	0.1183	0.0955	0.0405	0.132	0.0623	0.0531	0.0469	0.0616
10	0.1206	0.1120	0.1070	0.0503	0.1678	0.0788	0.0542	0.055	0.0706
15	0.1263	0.1302	0.1226	0.0467	0.1711	0.0756	0.0510	0.0601	0.1002
20	0.1267	0.1460	0.1378	0.0502	0.1711	0.0818	0.0552	0.0672	0.1206
25	0.1227	0.1433	0.1460	0.0518	0.1666	0.0915	0.0513	0.0645	0.1300
30	0.1163	0.1320	0.1464	0.0326	0.1670	0.0995	0.0640	0.0628	0.1333
35	0.1239	0.1323	0.1449	0.0318	0.1630	0.1016	0.0629	0.0572	0.1327
40	0.1224	0.1359	0.1488	0.0315	0.1653	0.0965	0.0679	0.0582	0.1154
45	0.1279	0.1357	0.1527	0.0422	0.1647	0.0973	0.0770	0.0538	0.1182
50	0.1344	0.1382	0.1606	0.0390	0.1686	0.0998	0.0828	0.0596	0.1149

**Table 14.** Mean average precision difference of nine term selection algorithms at 40 documents

No. ofterms	FR metrics								
	*^Δ^*Chi-square	*^Δ^*Chi	*^Δ^*DFC	*^Δ^*KLD	*^Δ^*RSV	*^Δ^*CoDice	*^Δ^*IG	*^Δ^*LRF	*^Δ^*PRF
5	0.0777	0.1074	0.1011	0.0496	0.1468	0.0696	0.0388	0.0436	0.0451
10	0.1042	0.1277	0.1329	0.0644	0.1648	0.0740	0.0412	0.0412	0.0761
15	0.1280	0.1167	0.1221	0.0444	0.1630	0.0738	0.0577	0.0452	0.1005
20	0.1146	0.1229	0.1321	0.0404	0.1616	0.0862	0.0578	0.0395	0.1260
25	0.1177	0.1261	0.1402	0.0354	0.1663	0.0883	0.0582	0.0384	0.1340
30	0.1241	0.1369	0.1504	0.0378	0.1586	0.0978	0.0551	0.0440	0.1362
35	0.1304	0.1396	0.1609	0.0390	0.1652	0.0958	0.0642	0.0374	0.1335
40	0.1201	0.1383	0.1610	0.0448	0.1718	0.0962	0.0658	0.0410	0.1376
45	0.1241	0.1385	0.1562	0.0479	0.1655	0.0983	0.0714	0.0474	0.1417
50	0.1238	0.1356	0.1613	0.0396	0.1648	0.0928	0.0630	0.0458	0.1430

**Table 15.** Mean average precision difference of nine term selection algorithms at 50 documents

No. ofterms	FR metrics								
	*^Δ^*Chi-square	*^Δ^*Chi	*^Δ^*DFC	*^Δ^*KLD	*^Δ^*RSV	*^Δ^*CoDice	*^Δ^*IG	*^Δ^*LRF	*^Δ^*PRF
5	0.083	0.0956	0.0901	0.0443	0.1319	0.0462	0.0069	0.0120	0.0436
10	0.0952	0.1179	0.1178	0.0431	0.1563	0.0648	0.0281	0.0409	0.0707
15	0.1227	0.1249	0.1231	0.0444	0.1558	0.0567	0.0340	0.0383	0.0935
20	0.1236	0.1302	0.1295	0.0383	0.1519	0.0571	0.0389	0.0246	0.1138
25	0.1197	0.1274	0.1306	0.0475	0.1573	0.0666	0.0369	0.0252	0.1254
30	0.1150	0.1297	0.1446	0.0533	0.1614	0.0772	0.0383	0.0183	0.1271
35	0.1195	0.1288	0.1503	0.0330	0.1593	0.0717	0.0363	0.0205	0.1294
40	0.1197	0.1326	0.1511	0.0359	0.1665	0.0736	0.0390	0.0275	0.1340
45	0.1226	0.1239	0.1566	0.0309	0.1675	0.0752	0.0491	0.0221	0.1385
50	0.1187	0.1217	0.1576	0.0316	0.1655	0.0762	0.0491	0.0271	0.1381

From above equation, we can deduce that having a very large value of Delta implies that the algorithm is affected by stemming in a negative way i.e. its performance has decreased majorly after applying stemming. On the other hand, least value of delta shows the small difference effect of stemming on algorithm i.e. performance of algorithm before and after stemming is almost same.

Table 11 illustrates the performance difference of term selection algorithms for 10 number of documents and terms *T* = 5, 10, 15, 20, …, 50. We can clearly observe that the overall performance of KLD is least affected by stemming. Chi and RSV are badly affected by stemming and have revealed very bad performance after stemming the dataset.

Table 12 highlights the difference in the performance of term selection algorithms for document =20 and terms= 5, 10, 15, …, 50. Largest values of deltas are obtained by RSV and DFC which shows high effect of stemming on these two algorithms. Opposite results are obtained by KLD once again as it has shown resistance toward stemming and its behavior after stemming stayed the same as before.

In Table 13, we have depicted the Deltas of nine term selection algorithms for 30 number of documents and pre-defined term test points (*T* = 5, 10, …, 50). KLD once again has refused to change its results after stemming and its precision before stemming are almost identical as it depicts least values of deltas at all term test points. However, at test point *T* = 25, IG has a very small value of delta which is almost comparable to the delta value obtained by KLD at same test point. Term selection algorithm that is most affected by stemming is RSV as it has the largest difference in precision value for all term test points.

Now in Tables 14 and 15 we observe the results on the algorithms before and after applying stemming on data using 40 and 50 documents, respectively, and varying the top expansion terms from 5 to 50 with a gap of 5 terms as 5, 10, 15, …, 50. A thorough inspection of the results mentioned in both tables illustrate that the performance of RSV is once again most affected by stemming. The precision of RSV obtained after stemming is much lower than the precision without stemming. At Document test point *D* = 40, results obtained by IG, LRF and KLD after stemming are almost same as before stemming. While in Table 15, only the precisions and results of IG and LRF are badly affected by stemming.

In conclusion, we can say that stemming in biological domain decreases the overall performance of term selection algorithms. RSV is very much vulnerable to the effect of stemming as its performance decreases the most after applying it on stemmed dataset. However, KLD has shown the most resistance against stemmed dataset and its precision before and after stemming stays almost same.

## Conclusion

We have proposed a new term selection algorithm named as ‘DFC’ for QE. DFC has been compared with other eight state-of-the-art term selection algorithms. Experiments show that DFC outperforms all other eight term selection algorithms in 88% of the pre-defined test points. DFC also caters the problem of document misclassification that occurs while setting the threshold of relevant and non-relevant class in pseudo relevance feedback. From Table 1 it can be concluded that chi-square has caused query drift for 25 of the total queries. On the other hand, DFC has shown an improvement in precision of 20 queries. To summarize the performance of all nine term selection algorithms, we have concluded that at defined set of document threshold (10, 20, 30, 40, 50), comparative performance of DFC is (60, 90, 90, 100, 100%). We also noticed that as the number of feedback document is increased, performance of DFC also increased while other term selection algorithms have marked an unexpected decrease. We would also like to mention that for PRF based information retrieval in biomedical domain, stemming tends to decrease the precision of all nine term selection algorithms.

*Conflict of interest*. None declared.
